# Possible Involvement of Standardized *Bacopa monniera* Extract (CDRI-08) in Epigenetic Regulation of *reelin* and Brain-Derived Neurotrophic Factor to Enhance Memory

**DOI:** 10.3389/fphar.2016.00166

**Published:** 2016-06-27

**Authors:** Jayakumar Preethi, Hemant K. Singh, Koilmani E. Rajan

**Affiliations:** ^1^Behavioral Neuroscience Laboratory, Department of Animal Science, School of Life Sciences, Bharathidasan UniversityTiruchirappalli, India; ^2^Laboratories for CNS Disorder, Learning and Memory, Division of Pharmacology, Central Drug Research InstituteLucknow, India

**Keywords:** *Bacopa monniera*, Novel object recognition, *reelin*, apolipoprotein E receptor 2 (*ApoER* 2), *N*-methyl-D-aspartate receptor (NMDAR), brain-derived neurotropic factor (BDNF)

## Abstract

*Bacopa monniera* extract (CDRI-08; BME) has been known to improve learning and memory, and understanding the molecular mechanisms may help to know its specificity. We investigated whether the BME treatment alters the methylation status of *reelin* and brain-derived neurotropic factor (BDNF) to enhance the memory through the interaction of *N*-methyl-D-aspartate receptor (NMDAR) with synaptic proteins. Rat pups were subjected to novel object recognition test following daily oral administration of BME (80 mg/kg) in 0.5% gum acacia (per-orally, p.o.; PND 15–29)/three doses of 5-azacytidine (5-azaC; 3.2 mg/kg) in 0.9% saline (intraperitoneally, i.p.) on PND-30. After the behavioral test, methylation status of *reelin*, BDNF and activation of NMDAR, and its interactions with synaptic proteins were tested. Rat pups treated with BME/5-azaC showed higher discrimination towards novel objects than with old objects during testing. Further, we observed an elevated level of unmethylated DNA in *reelin* and BDNF promoter region. Up-regulated *reelin* along with the splice variant of apolipoprotein E receptor 2 (*ApoER 2, ex 19*) form a cluster and activate NMDAR through disabled adopter protein-1 (DAB1) to enhance BDNF. Observed results suggest that BME regulate *reelin* epigenetically, which might enhance NMDAR interactions with synaptic proteins and induction of BDNF. These changes may be linked with improved novel object recognition memory.

## Introduction

Long-term memory formation requires fine tuned cellular signaling coordination with transcriptional and translational regulations of gene expression (Hawk and Abel, 2010). Epigenetic mechanisms specifically control transcription through many ways, among which DNA methylation being common (Levenson et al., 2006; Blaze and Roth, 2013; Morris and Monteggia, 2014). This process involves DNA methyltransferases (DNMTs) that mediates *de novo* methylation within DNA and alters the chromatin structure (Shilatifard, 2006; Lubin, 2011; Nabel and Kohli, 2011); which directly controls transcription. Several studies have reported that DNA methylation/demethylation in a specific promoter region of *reelin* and brain-derived neurotrophic factor (BDNF) dictates the transcriptional activity thereby critically regulating synaptic plasticity, learning, and memory (Miller and Sweatt, 2007; Levenson et al., 2008; Lubin et al., 2008; Mizuno et al., 2012; Sui et al., 2012). *Reelin* is a large secreted glycoprotein richly expressed at hippocampus by a subset of γ-amino butyric acid (GABA)-ergic interneurons (Pesold et al., 1998; Abraham and Meyer, 2003; Ramos-Moreno et al., 2006).

*Reelin* exerts its effects through two receptors: apolipoprotein E receptor 2 (*ApoER 2*) and very-low-density lipoprotein receptors (VLDLR) (Weeber et al., 2002; Strasser et al., 2004). *ApoER 2* expression in hippocampus promotes neuronal signaling and induction of long-term potentiation (LTP) (Barr et al., 2007). However, only the spliced variant of *ApoER 2* with exon 19 (ex 19) (Beffert et al., 2005) interacts with NPxY domain of adopter protein disabled-1 (DAB1) to elicit intracellular signaling (Howell et al., 1999; Benhayon et al., 2003; Beffert et al., 2006). Activation/phosphorylation of DAB1 (p-DAB1) by *reelin*-induced receptor cluster, depends on Src family tyrosine kinases (SFKs). The activated p-DAB1 in turn activates SFKs (Strasser et al., 2004; Kuo et al., 2005). Subsequently, activated SFKs phosphorylate the subunits of *N*-methyl-D-aspartate (NMDA; NR2A/NR2B) receptors (Yu et al., 1997; Chen et al., 2005; Mota et al., 2014). Further, the activated NMDA receptors target the activation of BDNF (Lubin et al., 2008). On the other hand, subunits of NMDA receptor directly interact with post-synaptic density protein-95 (PSD-95′ Hoe et al., 2006), and induces LTP and long-term memory (LTM).

*Bacopa monniera* extract has been traditionally used in Ayurvedic medicine to improve learning and memory. *B. monniera* extract contains triterpene saponins, which have been named bacosides and bacopasaponins. The major chemical entity shown responsible for neuropharmacological effects of *B. monniera* are bacoside A (bacogenins A1, A2, A3, and A4) and bacoside B (Pubmed C ID: 53398644); the latter differs only in optical rotation and may probably be an artifact produced during the process of isolating bacoside A (Chatterji et al., 1963, 1965; Basu et al., 1967). CDRI-08 mentioned as BME in this article is a bacoside-enriched standardized extract of *B. monniera* (CDRI-08, contains 55 ± 5% bacosides). Earlier studies demonstrate that CDRI-08 significantly improves the cognitive performance in healthy human participants (Downey et al., 2013; Benson et al., 2014); and in elders and patients with neurodegenerative disorder (Barbhaiya et al., 2008; Calabrese et al., 2008; Stough et al., 2013). Bacosides present in the CDRI-08 are non-polar glycosides, which possibly cross the blood–brain barrier (BBB) by lipid mediated passive diffusion (Pardridge, 1999); and its biodistribution in brain has been confirmed by radiopharmaceuticals (De et al., 2008). Earlier, in rat model, we found that oral treatment of BME elevates the level of serotonin (5-hydroxytryptamine, 5-HT), activates 5-HT_3A_ receptor (Rajan et al., 2011), and regulates cyclic adenosine monophosphate (cAMP) response element binding (CREB) protein through microRNA (miR)-124 (Preethi et al., 2012). Subsequently, we reported that BME regulates extracellular signal-regulated kinase (ERK)/CREB cascade and BDNF through regulation of histone acetylation and protein phosphatases to improve hippocampal memory (Preethi et al., 2014). In the present study, first we tested whether the treatment of BME/DNMT inhibitor (5-azacytidine and 5-azaC) improves novel object recognition. Second, we examined whether BME/5-azaC treatment alters methylation status of *reelin* and BDNF, and its effect on NMDA (NR2A) receptor interaction with synaptic proteins.

## Materials and Methods

### Animals

Wistar rat pups both male and female (*Rattus norvegicus*; B.wt: 19.6 ± 0.6 g on PND-15) were housed in a standard laboratory cage (43 × 27 × 15 cm) with paddy husk as a bedding material. Rat pups were maintained at animal house under controlled environmental condition [12-h light/dark cycle (7:00–19.00); temperature: 22 ± 2°C; humidity: 50 ± 5%)] with *ad libitum* access to food and water. Protocols for animal use were performed following the guidelines of Institutional Animal Ethics Committee (BDU/IAEC/2014/OE/08/Dt. 18.03.2014), ensuring that number of animals and pain was kept to a minimum.

### Drugs and Treatment

Standardized extract of *B. monniera* (CDRI-08 with 55 ± 5% bacosides) (Gifted by Lumen Marketing Company, Chennai, India), was dissolved in 0.5% gum acacia (Hi-Media Laboratories Pvt. Ltd, Mumbai, India). The DNA methyltransferase (DNMT) inhibitor, 5-azacytidine (5-azaC; ≥ 98%, Sigma–Aldrich, India) was dissolved in 0.9% saline. Drugs were prepared freshly on the day of administration (10:00–11:00 h) and drugs were administered to rat pups during their brain growth spurt period (Dobbing and Sands, 1979). Rat pups from different litters which attained the age of postnatal day (PND)-14 were randomly assigned into three groups: (i) control group (Con, *n* = 24) received vehicle solution 0.5% gum acacia, per-orally (p.o.) from PND 15–29 and three doses of 0.9% saline intraperitoneally (i.p.) on PND-30 (0, 5, and 23 h), (ii) BME group (BME, *n* = 24) received 80 mg/kg of CDRI-08 in 0.5% gum acacia (p.o.) from PND 15–29 (Rajan et al., 2011) and three doses of 0.9% saline (i.p.) on PND-30 (0, 5, and 23 h), (iii) 5-azaC group (5-azaC, *n* = 24) received 0.5% gum acacia (p.o.) from PND 15–29 and three doses of 3.2 mg/kg of 5-azaC (i.p.) on PND-30 (0, 5, and 23 h) (Sales et al., 2011).

### Novel-Object Recognition Test

The apparatus used for novel-object recognition (NOR) test was conducted in circular open-field board (82 cm diameter) made of wood, coated black surrounded by a wall of 32 cm height (Barbosa et al., 2013). Two sets of objects (Cuboids and Spherical objects) were used. The cuboids (13 × 10 × 10 cm) were made of plaster of paris and painted with blue color (non-toxic children water color). The plastic balls (radius: 26 cm) were green in color and filled with sand to ensure that animals cannot displace them. The apparatus and objects were cleaned with 75% ethanol after each behavior session. The sessions were recorded by a video camera (Sony megapixel 12.1, Full HD 1080) placed at a distance of 150 cm above the apparatus. In novel object recognition test, rodents’ innate habit of exploring novel object in their environment has been utilized to evaluate their memory (Ennaceur and Delacour, 1988). Exploration time was scored as when the rat nose was touching the object or orienting its head towards the object within a distance of 1 cm. The relative exploration time was recorded and expressed by a discrimination index [D.I. = (*t*_novel_ -*t*_old_)/(*t*_novel_ + *t*_old_) × 100%] as reported earlier (Stefanko et al., 2009). Mean exploration times were calculated and the discrimination indexes between groups were compared. Rat was allowed to freely explore the experimental area in groups (*n* = 3) on PND-28, 29 and individually on PND-30 for 10 min. On PND-31, all animals were trained for two sessions (5 min each session) with an interval of 1 h. During training, rats were exposed to four identical objects. On PND-32 during retention session, two new objects were placed in the position of any two old objects and the exploration behavior of each rat was recorded for 5 min (Barbosa et al., 2013).

### Tissue Collection, RNA and Protein Isolation

After the NOR test, animals representing each group (*n* = 6) were euthanized, and the hippocampus tissue was dissected as described by Glowinski and Iversen (1996) for the preparation of RNA and protein. Total RNA was isolated from hippocampus tissue samples using TRIzol (Merck Specialties Pvt. Ltd., India), according to the manufacturer’s instructions and stored with RNase inhibitor (Merck Specialties Pvt. Ltd, India) at -80°C. The hippocampus tissue samples were homogenized in ice-cold lysis buffer (150 mM NaCl, 50 mM Tris–HCl pH 7.5, 5 mM EDTA, 0.1% v/v NP-40, 1 mM DTT, 0.2 mM sodium orthovanadate, 0.23 mM PMSF) and 10 μl/ml protease inhibitor cocktail (Sigma–Aldrich, USA). The homogenates were kept on ice for 30 min and then centrifuged at 10,000 × g for 30 min at 4°C. The clear supernatants were collected in a fresh tube and centrifuged again at 12,000 × g for 15 min at 4°C. The final supernatants were collected and stored as aliquots, in order to avoid repeated freeze–thaw of the samples and were stored at -80°C. The concentration of each protein sample was quantified by measuring the absorbance at 595 nm using Biophotometer plus (Eppendorf Inc., Germany).

### DNA Methylation Assay

Individuals representing each group (*n* = 6) were euthanized and DNA was isolated from hippocampus tissue (UltraClean^®^ Tissue & Cells DNA isolation kit, Cat no. 12334-50, MO BIO laboratories, Inc., USA) and processed for bisulfite modification (EpiTect Bisulfite kit, Qiagen, Cat no. 59104).

### Quantitative Real-Time PCR (qRT-PCR)

The qRT-PCR was performed using real-time reaction mixture (SSoAdvanced^TM^ SYBR^®^ green supermix, Bio-Rad Laboratories, Inc., USA) with specific primers (50 pmol/μl) and DNA (0.4 ng/μl)/ primers (200 pmol/μl) and cDNA (0.2 μg/μl) in CFX-96 Touch^TM^ Real-time PCR (RT-PCR) Detection System (Bio-Rad Laboratories, Inc., USA).

The detection of unmethylated DNA in the gene promoter was performed using the specific primers for *reelin* (For 5′-TGTTAAATTTTTGTAGTATTGGGGATGT-3′; Rev 5′-TCCTTA AAATAATCCAACAACACACC-3′) and BDNF (CpG island 1) (For 5′-GGGTAGTGATTTT GGGGAGGAAGTAT-3′; Rev 5′-CAACCTCTATACACAACTAAATCCACC-3′) (Sui et al., 2012). Reactions were performed with the following cycling conditions: 95°C for 3 min, 40 cycles of 95°C for 15 s, 62.6°C (*reelin*)/65.5°C (BDNF) for 1 min and 73.5°C (*reelin*)/72 °C (BDNF) for 15 s. Detection of methylated DNA was performed using specific primers for *reelin* (For 5′-GGTGTTAAATTTTTGTAGTATTGGGGAC-3′; Rev 5′-TCCTTAAAATAATCCAA CAACACGC-3′) and CpG island 1 of BDNF (For 5′-GTA GCGATTTTGGGGAGGAAGTAC-3′; Rev (5′-CAACCTCTATACGCGACTAAATCCG-3′) (Sui et al., 2012). Reactions were performed with the following cycling conditions: 95°C for 3 min, 40 cycles of 95°C for 15 s, 60°C (*reelin*)/61°C (BDNF) for 1 min and 72°C (*reelin*)/74°C (BDNF) for 15 s. To verify specificity, the final product was electrophoresed on 2% agarose gel stained with ethidium bromide (0.5 μg/ml), and amplification and band intensity was examined in ChemiDoc XR + System with image lab 2 (Bio-Rad Laboratories, Inc., USA).

To examine the expression level of mRNA, total RNA (2.0 μg/sample) was reverse transcribed into cDNA using random/oligo-dT primers (iScript cDNA Synthesis Kit, Bio-Rad Laboratories Inc., USA). The expression level of specific genes were estimated using specific primers *reelin* (For: 5′-AAACTACAGCGGGTGGAACC-3′ and Rev: 5′-ATTTGAGGCATGA CGGACCTATAT-3′) (Sui et al., 2012)/BDNF (For: 5′-GTAAACGTCCACGGACAAG-3′ and Rev: 5′-TATGGTTTTCTTCGTTGGGC-3′), Total *ApoER 2* (For: 5′-AGTGTCCCGATGGC TCTGAC-3′ and Rev: 5′-CAGCTTAACTTCTCGGCAGGA-3′), *ApoER 2 (ex19)* For: 5′GCC CTCAAGGAGCTTTTTGTC-3′ and Rev: 5′-AGGGTTCTTCGGGAGTTGGT-3′), *ApoER 2* (*Δ*) For: 5′-CAGTGTACAGGAAAACGACAGAAGA-3′ and Rev: 5′-TGCCACTCGTGCG GG-3′ (Beffert et al., 2005) with Glyceraldehyde 3-phosphate dehydrogenase (GAPDH) (For: 5′-AACATCATCCCTGCATCCAC-3′ and Rev: 5′-AGGAACACGGAAGGCCATGC-3′) as internal control. Conditions for the RT-PCR reactions were as follows: initial denaturation at 94°C for 3 min followed by denaturation at 94°C for 5 s, annealing (64.5°C for *reelin*, 60°C for BDNF, 55°C for total *ApoER 2*, 60°C for *ApoER 2 (ex19)*, 57°C for *ApoER 2* (*Δ*) and 56°C for GAPDH) for 5 s, extension at 72°C for 5 s. The amplification of the single PCR product was confirmed by monitoring the dissociation curve followed by melting curve analysis. Each reaction was performed in triplicates and normalized with internal control GAPDH. The data were presented as mean fold change relative to the control group (CFX Manager^TM^ version 2 software; CFX-96 Touch^TM^ RT-PCR Detection System Bio-Rad Laboratories, Inc., USA).

### Western Blot Analysis

Equal concentrations (30 μg) of proteins were resolved on 9% SDS–polyacrylamide gel. The separated proteins were transferred electrophoretically onto polyvinylidene difluoride (PVDF) membrane (Merck Millipore, India) using a semi-dry western apparatus (SD 20; Cleaver Scientific Ltd, UK). The membranes were blocked and incubated at 4°C for 9 h with one of the following specific primary antibodies (Santa-Cruz Biotechnology, Cell signaling solutions, USA): rabbit polyclonal anti-BDNF (N-20) [SC-546, 1:500], anti-PSD-95 [SC-28941, 1:200], anti-Dab1 [Cat #3328, 1:1000], anti-Phospho-Dab1 [Cat #3327, 1:1000], anti-NMDAR2A [Cat #4205, 1:1000], anti-NMDAR2B [Cat #4207, 1:1000] antibodies, mouse monoclonal anti-p-Tyr antibody [SC-7020, 1:300] and rabbit polyclonal anti-β-actin antibody (SC-130656; 1:200) was used as control for each sample. The membrane-bound antibodies were detected by incubating for 3 h at 4°C with alkaline phosphatase (ALP) conjugated either with goat anti-rabbit (Cat #621100180011730, MERCK, 1:2000), goat anti-mouse (Cat #621100480011730, MERCK, 1:2000) secondary antibody. Subsequently alkaline phosphatase activity was detected with 5-bromo-4-chloro-3-indolyl phosphate di-sodium salt (BCIP)/nitroblue tetrazolium chloride (NBT) (HIMEDIA, Mumbai, India) according to the manufacturer’s instructions. Images were acquired with a ChemiDoc XR + System (Bio-Rad Laboratories, Inc., USA), and optical density of trace quantity for each band was measured using Image Lab 2 software (Bio-Rad Laboratories, Inc., USA). The trace quantity of each bands were normalized with β-actin bands, following that fold changes were calculated by dividing normalized values of BME/5-azaC groups by control groups.

### Co-immunoprecipitation

Spin columns were packed with AminoLink Plus Coupling resin slurry (50 μl) and washed with provided buffer, then 20 μg of anti-rabbit NR2A antibody (Cat #4205, adjusted to 200 μl volume with 1× coupling buffer) was added in the column and incubated on a rotator at room temperature for 2 h to immobilize antibodies. For Co-IP, 200 μg of protein lysates from each groups Con/BME/5-azaC were immunoprecipitated with NR2A antibody in immobilized spin columns overnight at 4°C. Immunoprecipitated protein was eluted using elution buffer. The complete Co-IP experiment performed following the instructions provided with the kit (Catlogue no. 26149, Pierce Co-Immunoprecipitation Kit, Thermofisher, IL, USA). Equal volume of NR2A precipitated protein from each sample elutes was analyzed by immunoblotting using a SFK (SC-7020, 1:300) and PSD-95 (SC-28941, 1:200) antibody. Immunoblotting was performed following the procedure reported in the western blots section.

### Statistics

Data were presented as a mean ± standard error of the mean (SEM) and plotted with KyPlot (ver 1.0) for graphical representation. For behavioral analysis (NOR), multivariate ANOVA was performed to test the effect of factors (training sessions, objects) and their interactions during training. One-way analysis of variance (ANOVA) and Two-way analysis of variance (Two-way ANOVA) were used to assess the significance between groups and groups × objects interactions during testing respectively. *Post hoc* Bonferroni test was used to examine the difference between groups. Association between the recognition memory (DI) and methylation/unmethylation DNA was estimated using Pearson correlations (SPSS, ver.15). For expression data, One-way analysis of variance (ANOVA) was used to observe the significance between groups. Differences were considered significant if *P* < 0.05.

## Results

### BME Improves Novel Object Recognition

**FIGURE 1:**
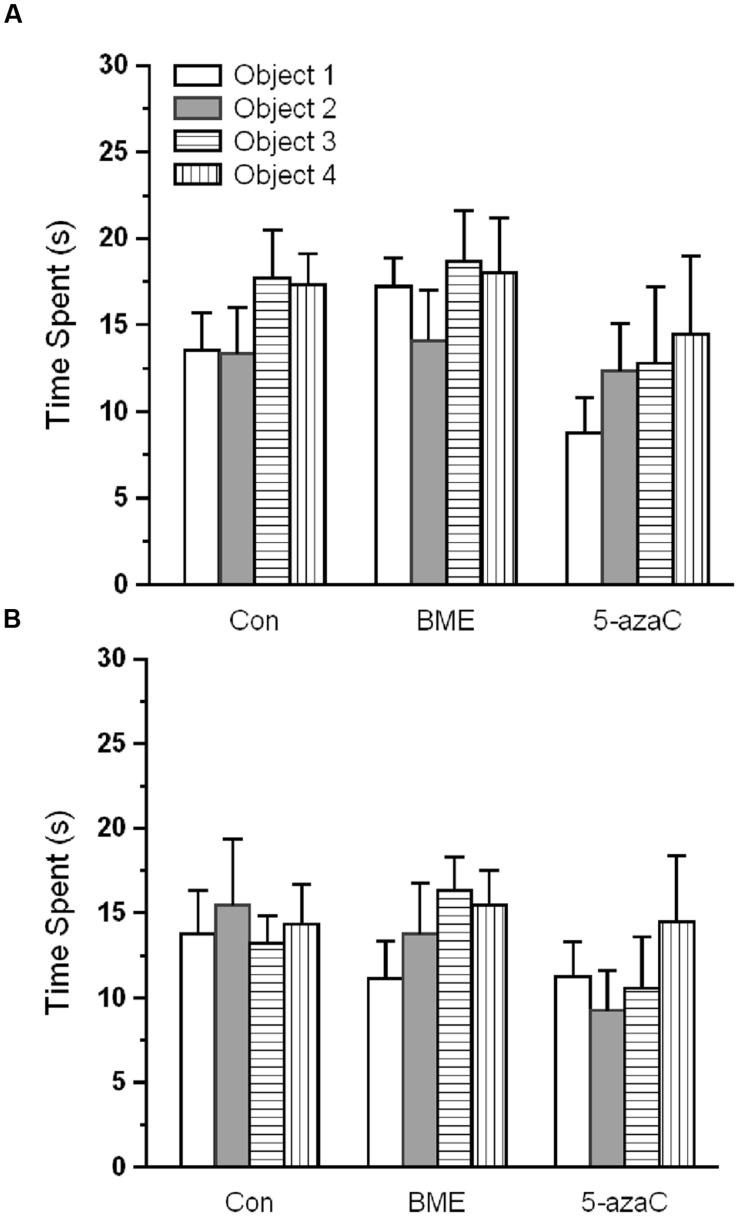
**Effect of BME/5-azaC on training at novel object recognition test.** **(A)** Con/BME/5-azaC groups spent equal time and showed no discrimination for objects during training 1, **(B)** and training 2. Data were shown as mean ± SEM, there was no significant difference between comparisons (Con verses BME; Con verses 5-azaC; BME verses 5-azaC).

**FIGURE 2:**
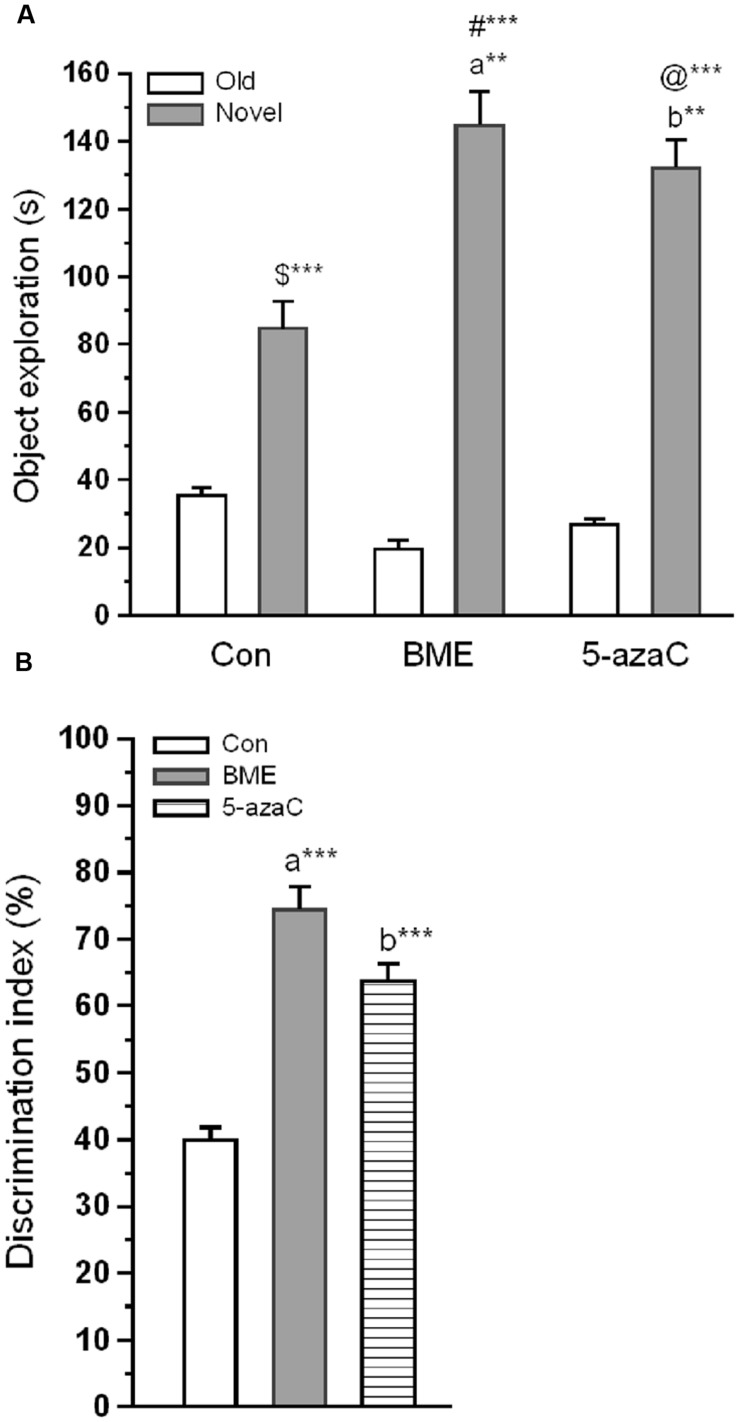
**Effect of BME/5-azaC on novel object recognition test.** **(A)** Con/BME/5-azaC group rats spent more time(s) around novel object than old objects. Whereas, BME/5-azaC groups spent significantly more time around novel object compared to control group. **(B)** BME/5-azaC treated groups displayed significantly higher DI (%) for novel object compared to control group. Data were shown as mean ± SEM, asterisk indicates significant difference (^∗∗^*P* < 0.01; ^∗∗∗^*P* < 0.001). Comparisons between groups are represented as a = Con verses BME; b = Con verses 5-azaC; and c = BME verses 5-azaC; $ = Con old verses novel; # = BME old verses novel; @ = 5-azaC old verses novel.

Novel object recognition test was performed to examine the effect of BME on hippocampus dependent long-term memory. At first, rat pups from experimental groups (Con, BME, 5-azaC) were subjected to training. Multivariate ANOVA analysis revealed that there was no significant difference between training sessions [Wilks’ *λ* = 0.953; *F* = 1.016; *P* = 0.392], objects [Wilks’ *λ* = 0.916; *F* = 0.614; *P* = 0.784] and interaction of training sessions × objects [Wilks’ *λ* = 0.9356; *F* = 0.466; *P* = 0.895]. Further, our analysis showed that the time spent by rat pups exploring the objects did not differ significantly between training sessions [Con: *F*_(1,71)_ = 0.493, *P* = 0.493; BME: *F*_(1,71)_ = 2.48, *P* = 0.120; 5-azaC: *F*_(1,71)_ = 0.096, *P* = 0.758], objects [Con: *F*_(3,71)_ = 0.295, *P* = 0.829; BME: *F*_(3,71)_ = 0.995, *P* = 0.401; 5-azaC: *F*_(3,71)_ = 0.691, *P* = 0.561] and interaction of training sessions × objects [Con: *F*_(3,71)_ = 0.672, *P* = 0.572; BME: *F*_(3,71)_ = 0.446, *P* = 0.721; 5-azaC: *F*_(3,71)_ = 0.284, *P* = 0.837] (**Figures 1A,B**; **Supplementary Table S1**). During retention test, Con/BME/5-azaC groups spent significantly more time around novel objects [Con: *F*_(2,17)_ = 31.825, *P* < 0.001; BME: *F*_(2,17)_ = 109.794, *P* < 0.001; 5-azaC: *F*_(2,17)_ = 116.415, *P* < 0.001] than old objects. Whereas, BME [*F*_(2,17)_ = 15.399, *P* < 0.01]/ 5-azaC [*F*_(2,17)_ = 11.681, *P* < 0.01] group rat pups spent significantly more time around novel objects than control group. Further two-way ANOVA revealed that there was a significant difference across the treatment groups [*F*_(2,53)_ = 5.279, *P* < 0.01] and objects [*F*_(1,53)_ = 245.476, *P* < 0.001] in novel object exploration time, and treatment groups × objects interaction [*F*_(2,53)_ = 12.43, *P* < 0.001]. In addition, Bonferroni *post hoc* test confirmed that the novel object exploration time was statistically higher in BME (*P* < 0.05) and 5-azaC (*P* < 0.05) compared to control (**Figure 2A**). BME/5-azaC treated group spent more time to explore the novel objects compared to old objects. Discrimination indexes were greater than zero for the study groups, where BME [*F*_(1,17)_ = 36.56, *P* < 0.001] and 5-azaC [*F*_(1,17)_ = 21.648, *P* < 0.001] group showed significantly higher DI than control group. While comparing BME and 5-azaC, there was no significant difference between them [*F*_(1,17)_ = 4.985, *P* = 0.040] (**Figure 2B**). Our results demonstrated that BME treatment effectively establish long-term memory for the familiar object, which was comparable to 5-azaC treatment.

### Effect of BME on Methylation of *Reelin* Promoter and Its Expression

**FIGURE 3:**
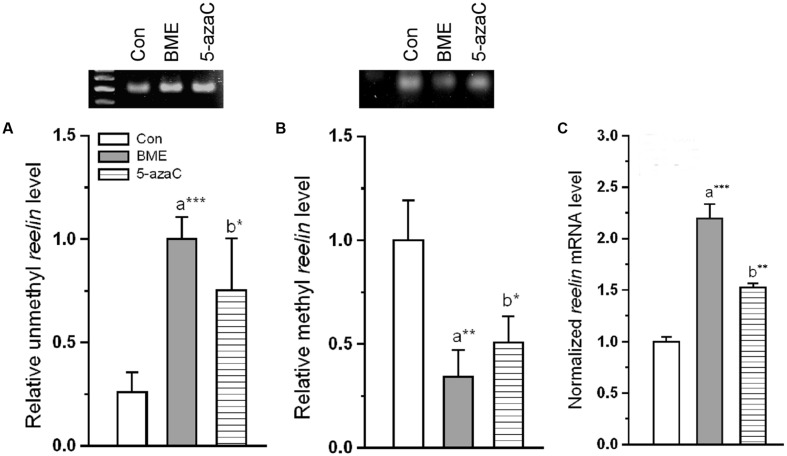
**Effect of BME/5-azaC on regulation of DNA methylation in the *reelin* promoter and *reelin* expression.** BME/5-azaC treatment significantly **(A)** increased unmethylated DNA, **(B)** and decreased methylated DNA in *reelin* promoter region relative to control group. **(C)** *reelin* mRNA was significantly higher in BME/5-azaC treated groups compared to control group. Data were shown as mean ± SEM, asterisk indicates significant difference (^∗^*P* < 0.05; ^∗∗^*P* < 0.01; ^∗∗∗^ *P* < 0.001). Comparisons between groups are represented as a = Con verses BME; b = Con verses 5-azaC; and c = BME verses 5-azaC.

To test whether the improvement in NOR by methylation status of *reelin* is due, atleast in part by BME treatment, we determine the effect of BME on methylation status of CpG island present in *reelin* promoter. We found that BME treatment induced a significant effect on methylation status of *reelin* and its expression. Our analysis showed that significant increase in unmethylated *reelin* DNA levels associated with BME [*F*_(1,5)_ = 78.132, *P* < 0.001] and 5-azaC treatment [*F*_(1,5)_ = 12.706, *P* < 0.05] than control. There were no changes in the unmethylation level of reelin DNA between BME and 5-azaC groups [*F*_(1,5)_ = 0.667, *P* = 0.460] (**Figure 3A**). Simultaneously, the level of methylated reelin DNA decreased in BME [*F*_(1,5)_ = 30.867, *P* < 0.01] and 5-azaC [*F*_(1,5)_ = 18.16, *P* < 0.05] compared to control group. Whereas, there was no significant difference between BME and 5-azaC groups [*F*_(1,5)_ = 5.280, *P* = 0.083] (**Figure 3B**). Interestingly, demethylation of *reelin* promoter resulted in significant up-regulation of *reelin* expression in BME [*F*_(1,11)_ = 28.79, *P* < 0.001] and 5-azaC groups [*F*_(1,11)_ = 17.98, *P* < 0.01] compared to control group, but there was no significant difference between BME and 5-azaC groups [*F*_(1,11)_ = 4.299, *P* = 0.065] (**Figure 3C**). Subsequently, we found a significant positive correlation between recognition memory (discrimination index; DI) and unmethylated *reelin* DNA (BME: *r* = 0.973, *P* < 0.001; 5-azaC: *r* = 0.878, *P* < 0.01), and a negative correlation between DI and methylated *reelin* DNA (BME: *r* = -0.965, *P* < 0.001; 5-azaC: *r* = -0.885, *P* < 0.01) in hippocampus. Overall, these data supported the idea that BME/5-azaC treatment possibly regulated the methylation of *reelin* promoter, as there was a correlation of decreased methylation with increased mRNA level.

### Effect of BME on Splicing of ApoER 2 Receptor

**FIGURE 4:**
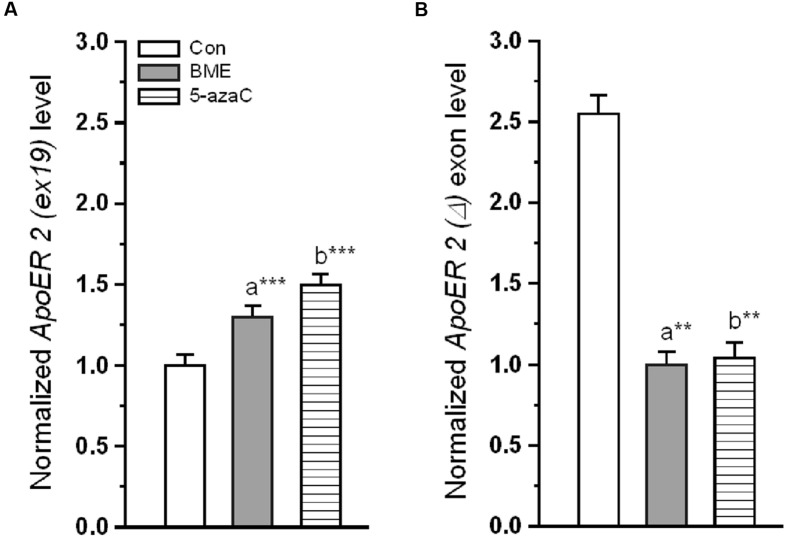
**Effect of BME/5-azaC on *ApoER 2* splicing of variants.** **(A)** BME/ 5-azaC treatment significantly increased the level of *ApoER 2 (ex 19)* mRNA, **(B)** and decreased levels of *ApoER 2 (Δ)* exon mRNA compared to control group. Data were shown as mean ± SEM, asterisk indicates significant difference (^∗∗^*P* < 0.01; ^∗∗∗^*P* < 0.001). Comparisons between groups are represented as a = Con verses BME; b = Con verses 5-azaC; and c = BME verses 5-azaC.

One could expect that, the altered *reelin* could contribute to the possible changes on the target molecules. Alternative splicing of *ApoER 2* was a critical component in the *reelin* mediated NMDA receptor activity. *ApoER 2 (ex 19)* mRNA (**Figure 4A**) significantly increased in BME [*F*_(1,11)_ = 23.31, *P* < 0.001] and 5-azaC group [*F*_(1,11)_ = 31.28, *P* < 0.001] relative to control group. Whereas, there was no significant difference between BME and 5-azaC groups [*F*_(1,11)_ = 0.832, *P* = 0.383]. Conversely, the *ApoER 2 (Δ) ex 19* mRNA variants were at lower level in BME [*F*_(1,11)_ = 19.35, *P* < 0.001] and 5-azaC groups [*F*_(1,11)_ = 13.2, *P* < 0.01] compared to control group, but there was no significant difference between BME and 5-azaC groups [*F*_(1,11)_ = 0.0016, *P* = 0.969] (**Figure 4B**). Up-regulated level of *ApoER 2 (ex 19)* may be linked with recorded improvement in NOR, suggesting that *ApoER 2 (ex 19)* splicing possibly induced by BME treatment.

### Effect of BME on *Reelin* Mediated NMDA in Phosphorylation of DAB1

**FIGURE 5:**
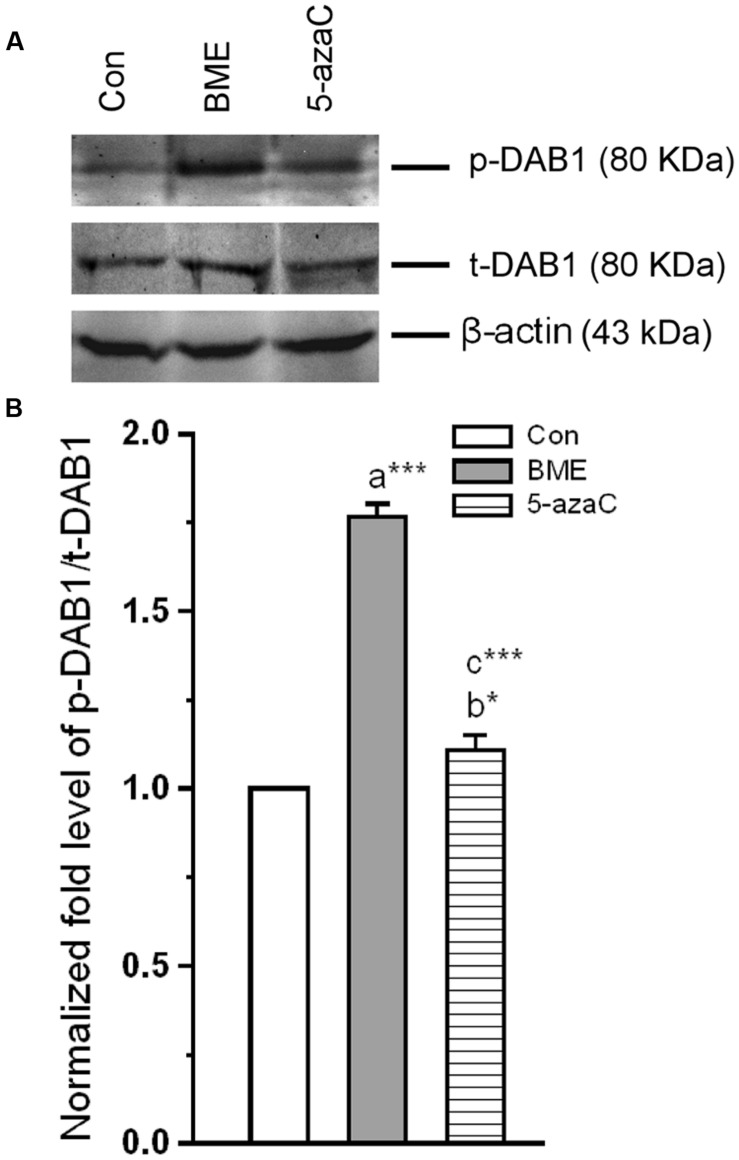
**Effect of BME/5-azaC on induction and phosphorylation of DAB1 in hippocampus.** **(A)** Western blots showing the immunoreactivity to anti-total DAB1, and anti-phosphorylated DAB1. **(B)** BME/5-azaC treatment increased levels of p-DAB1 compared to control. Data were shown as mean ± SEM, asterisk indicates significant difference (^∗^*P* < 0.05; ^∗∗∗^*P* < 0.001). Comparisons between groups are represented as a = Con verses BME; b = Con verses 5-azaC; and c = BME verses 5-azaC.

*Reelin* binding to *ApoER 2 (ex 19)* and VLDLR results in activation of DAB1. To expand our understanding, we tested whether the BME induced up-regulated *ApoER 2 (ex 19)* facilitates the phosphorylation of DAB1 (p-DAB1). Total and p-DAB1 level in Con/BME/5-azaC groups were determined. **Figures 5A,B** showed that BME/5-azaC treatment significantly increased the level of p-DAB1 in both BME [*F*_(1,5)_ = 150.662, *P* < 0.001] and 5-azaC [*F*_(1,5)_ = 14.844, *P* < 0.05] compared to control group. The level of p-DAB1 was higher in BME [*F*_(1,5)_ = 101.637, *P* < 0.001] than 5-azaC group. These results suggested that increased *ApoER 2 (ex19)* by BME/5-azaC was responsible for elevated levels of total and p-DAB1.

### Effect of BME on Alternation of NMDA Receptors

**FIGURE 6:**
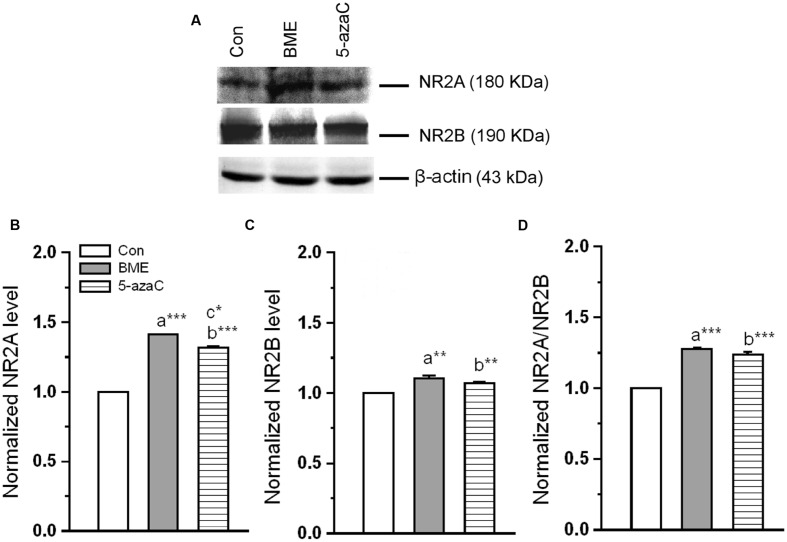
**Effect of BME/5-azaC on level of NMDAR subunits.** **(A)** Western blots showing the immunoreactivity to anti-NR2A, anti-NR2B. **(B)** BME/5-azaC treated groups showed comparatively increased level of NR2A, **(C)** and NR2B. **(D)** NR2A/NR2B ratio was significantly more in BME/5-azaC groups than control group. Data were shown as mean ± SEM, asterisk indicates significant difference (^∗^*P* < 0.05; ^∗∗^*P* < 0.01; ^∗∗∗^*P* < 0.001). Comparisons between groups are represented as a = Con verses BME; b = Con verses 5-azaC; and c = BME verses 5-azaC.

We examined whether the BME treatment altered the NMDARs subunits composition (**Figure 6A**). Western blot analysis showed that the level of NR2A was significantly higher in BME [*F*_(1,5)_ = 107.05, *P* < 0.001] and 5-azaC [*F*_(1,5)_ = 209.707, *P* < 0.001] compared to control group. While comparing BME with 5-azaC, the level of NR2A in BME [*F*_(1,5)_ = 12.128, *P* < 0.05] was higher than 5-azaC group (**Figure 6B**). Similarly, the level of NR2B was significantly higher in BME [*F*_(1,5)_ = 42.446, *P* < 0.01] and 5-azaC [*F*_(1,5)_ = 12.99, *P* < 0.05] compared to control group. Whereas, there was no significant difference between BME and 5-azaC groups [*F*_(1,5)_ = 6.931, *P* = 0.058] (**Figure 6C**). Therefore, the NR2A/NR2B ratio was significantly higher in BME [*F*_(1,5)_ = 100.439, *P* < 0.001] and 5-azaC [*F*_(1,5)_ = 103.83, *P* < 0.001] compared to control group. Whereas, there was no difference between BME and 5-azaC groups [*F*_(1,5)_ = 2.919, *P* = 0.163] (**Figure 6D**). Thus BME/5-azaC treatment increased the NMDAR (NR2) subunits and NR2A/NR2B ratio.

### Effect of BME on Interaction of NMDAR Subunit with Synaptic Proteins

**FIGURE 7:**
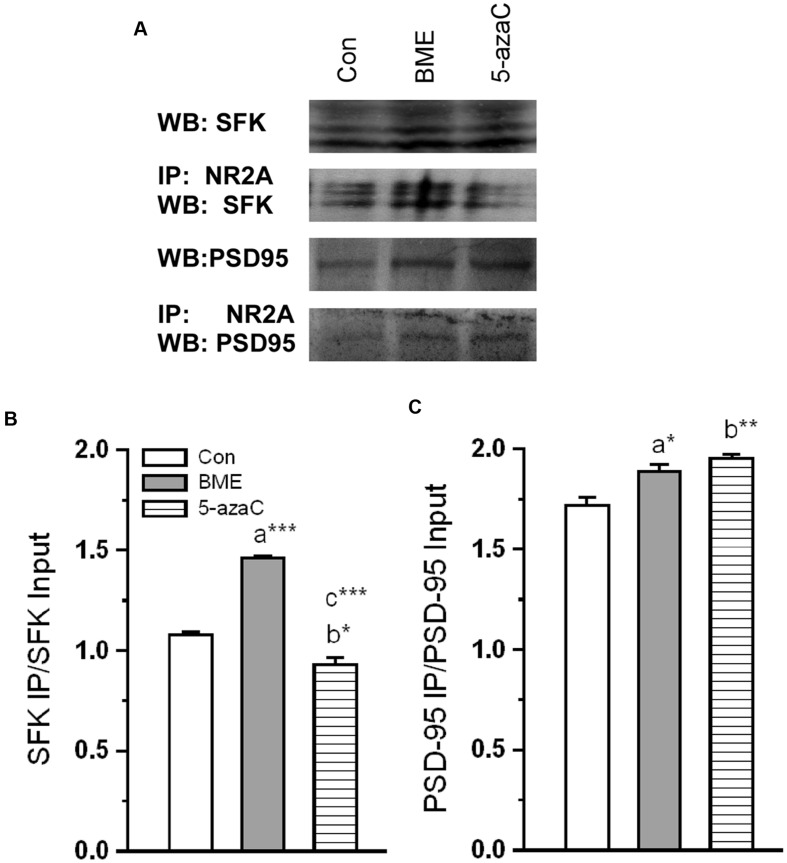
**Effect of BME/5-azaC on interaction of NR2A with synaptic proteins.** **(A)** Western blots showing immunoreactivity to anti-SFK and anti-PSD-95 in NR2A immunoprecipitated protein. **(B)** BME/5-azaC treatment significantly increased interaction of NR2A with SFK, **(C)** and PSD-95. Data were shown as mean ± SEM, asterisk indicates significant difference (^∗^*P* < 0.05; ^∗∗^*P* < 0.01; ^∗∗∗^*P* < 0.001). Comparisons between groups are represented as a = Con verses BME; b = Con verses 5-azaC; and c = BME verses 5-azaC.

Further, our Co-IP showed that observed alternation in NR2A level concomitantly enhanced interaction with key synaptic proteins (**Figure 7A**). The analysis revealed that the level of NR2A–SFK proteins was significantly higher in BME [*F*_(1,5)_ = 736.071, *P* < 0.001] than control group. Surprisingly, it was lower in 5-azaC group compared to control [*F*_(1,5)_ = 14.928, *P* < 0.05] and BME [*F*_(1,5)_ = 202.34, *P* < 0.001] (**Figure 7B**). While considering the level of interacted NR2A–PSD-95 proteins, it was significantly higher in both BME [*F*_(1,5)_ = 8.44, *P* < 0.05] and 5-azaC treated group [*F*_(1,5)_ = 27.079, *P* < 0.01] than the control group. Whereas, there was no significant difference between BME and 5-azaC [*F*_(1,5)_ = 4.56, *P* = 0.100] (**Figure 7C**). The higher level of SFK/PSD-95 with NR2A in BME/5-azaC groups, further supported NMDAR activation.

### Effect of BME Mediated NMDA Activation on Methylation and Expression of BDNF

**FIGURE 8:**
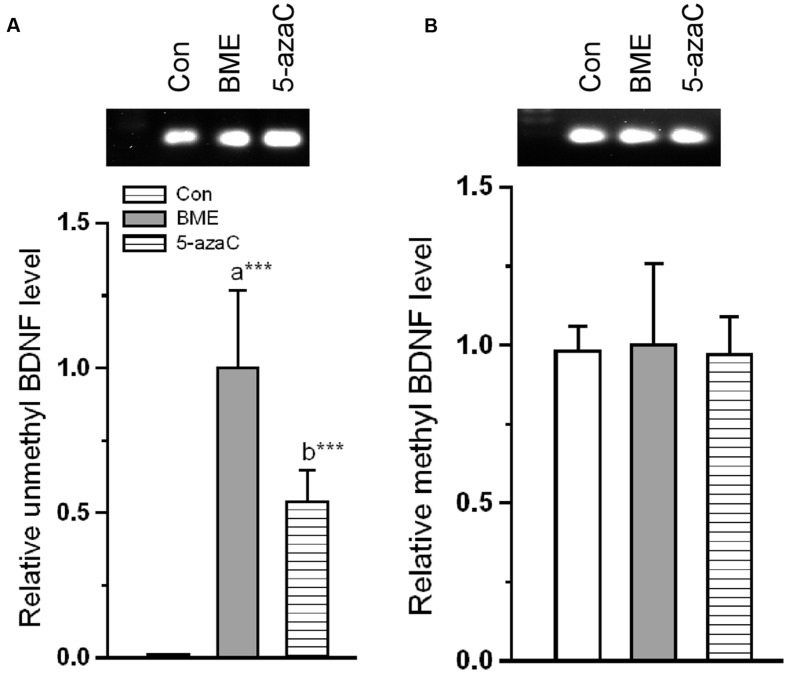
**Effect of BME/ 5-azaC on DNA methylation of BDNF promoter region.** **(A)** BME/ 5-azaC treatment increased unmethylated DNA in BDNF promoter region, **(B)** but did not alter the methylated BDNF. Data were shown as mean ± SEM, asterisk indicates significant difference (^∗∗∗^*P* < 0.001). Comparisons between groups are represented as a = Con verses BME; b = Con verses 5-azaC; and c = BME verses 5-azaC.

Further, we tested whether altered NMDAR interactions were correlated with transcriptional and translational activity of BDNF. First, we tested the level of methylation status of BDNF promoter. We found that the level of unmethylated BDNF was significantly increased in BME [*F*_(1,5)_ = 592.189, *P* < 0.001] and 5-azaC [*F*_(1,5)_ = 736.074, *P* < 0.001] groups compared to control (**Figure 8A**). But there was no significant difference between BME and 5-azaC groups [*F*_(1,5)_ = 3.438, *P* = 0.137]. However, the estimated methylated DNA in BME [*F*_(1,5)_ = 0.00181, *P* = 0.968] and 5-azaC [*F*_(1,5)_ = 0.335, *P* = 0.594] groups was not significantly different from control. Similarly, there was no significant difference between BME and 5-azaC groups [*F*_(1,5)_ = 0.196, *P* = 0.681] (**Figure 8B**). In addition, our analysis showed a significant positive correlation between DI and unmethylated BDNF DNA (BME: *r* = 0.970, *P* < 0.001; 5-azaC: *r* = 0.898, *P* < 0.01). We did not find a significant correlation between DI and methylated BDNF DNA (BME: *r* = 0.034, *P* = 0.936; 5-azaC: *r* = -0.460, *P* = 0.251) in hippocampus.

**FIGURE 9:**
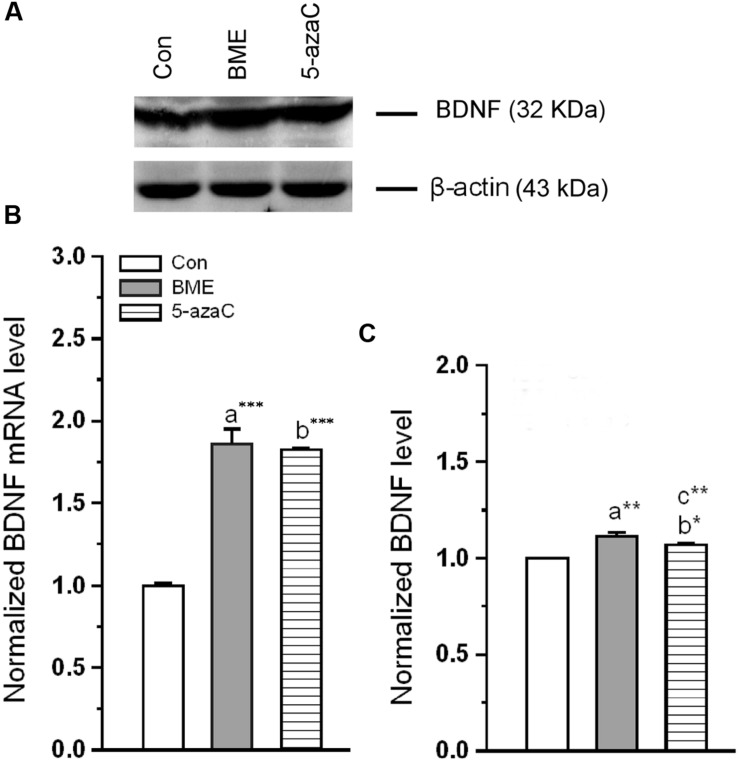
**Effect of BME/5-azaC on expression of BDNF mRNA and protein.** **(A)** Western blot showing immunoreactivity to anti-BDNF. **(B)** BME/5-azaC treatment increased BDNF mRNA levels, **(C)** and BDNF protein. Data were shown as mean ± SEM, asterisk indicates significant difference (^∗^*P* < 0.05; ^∗∗^*P* < 0.01; ^∗∗∗^*P* < 0.001). Comparisons between groups are represented as a = Con verses BME; b = Con verses 5-azaC; and c = BME verses 5-azaC.

Subsequently, we found that level of BDNF mRNA was significantly increased in BME [*F*_(1,5)_ = 119.153, *P* < 0.001] and 5-azaC treated groups [*F*_(1,5)_ = 342.48, *P* < 0.001] compared to control group. But there was no difference between BME and 5-azaC [*F*_(1,5)_ = 0.126, *P* = 0.73] (**Figure 9B**). Supporting to this, the western blot analysis showed that the level of BDNF protein was significantly higher in BME [*F*_(1,5)_ = 48.527, *P* < 0.01] and 5-azaC [*F*_(1,5)_ = 25.766, *P* < 0.01] compared to control group, but there was no significant difference between BME and 5-azaC [*F*_(1,5)_ = 1.866, *P* = 0.244] (**Figures 9A,C**). These data supported that the NMDAR activation by BME/5-azaC treatment altered the unmethylated BDNF levels and its expression.

## Discussion

Earlier studies demonstrated that DNA methylation/demethylation involves in the regulation of activity dependent neuronal gene expression (Ming and Song, 2011; Baker-Andresen et al., 2013) and synaptic plasticity (Miller and Sweatt, 2007; Feng et al., 2010). A family of DNMTs (DNMT1, 3a, 3b) can catalyze the process of DNA methylation (Sweatt, 2009). The expression pattern of DNMTs (DNMT1, 3a, 3b) differ in different brain regions following behavioral training and exhibits different behavioral phenotype (Feng et al., 2010; Miller et al., 2010; Morris et al., 2014). In fact, DNMT inhibitors (5-azaC) are known to alter learning and memory/memory consolidation (Miller and Sweatt, 2007). Considering the role of DNA methylation in memory formation, first we showed the effect of BME in novel object recognition by comparing with DNMT inhibitor, 5-azaC. Using NOR test, we found that BME effectively enhanced the NOR memory, this suggested that BME treatment improved the process of recollection and familiarity identification (Squire et al., 2007). It has been shown that methylation status alters rapidly and dynamically within hippocampus (Miller and Sweatt, 2007). The level of DNMT 3a was also significantly elevated following contextual training in NOR test, but its conditional knock-out impaired NOR memory (Morris et al., 2014). Observed behavioral results suggested that BME/5-azaC treatment might optimally regulate the DNMT 3a level at this age of rats and hence improve NOR memory.

Following the improvement in behavioral phenotype; we sought to correlate this with methylation status. As quantified by RT-PCR, a higher level of unmethylated and lower level of methylated *reelin* DNA was estimated in BME and 5-azaC treated groups when compared to control. Supporting this, earlier studies have shown that 5-azaC treatment reduces the methylated DNA in *reelin* promoter (Miller and Sweatt, 2007; Sui et al., 2012). Consistent with the demethylation status, the level of *reelin* mRNA was significantly elevated in both BME and 5-azaC treated rats. The positive correlation between recognition memory (DI) and unmethylated *reelin* DNA in BME/5-azaC group suggested that BME possibly regulated methylation to enhance memory. These results are in line with earlier reports on methylation status and expression of *reelin* during neural plasticity (Levenson et al., 2006; Miller and Sweatt, 2007). In addition, observed behavioral data may be linked with the up-regulated *reelin* expression (Rogers et al., 2011) and increased dendritic spine density in hippocampus (Vollala et al., 2011).

*Reelin* exerts its effects through *ApoER 2 ex 19* receptor (Weeber et al., 2002; Beffert et al., 2005). Interestingly, BME/ 5-azaC treatment up-regulated the *ApoER 2 (exon 19)* variant and down-regulated *ApoER 2(Δ)* transcript. The up-regulated *ApoER 2 exon 19* attaches to postsynaptic density protein PSD-95 and forms a signaling complex with NMDARs (Beffert et al., 2005; Hoe et al., 2006). However, to execute the neuronal transmission, NMDAR should be activated by p-DAB1 (Chen et al., 2005; Beffert et al., 2006). Consistently, BME/5-azaC treatment enhanced the level of p-DAB1. In process p-DAB1 activates Src kinase family (SFK) through phosphorylation, which in turn leads to phosphorylation of NMDAR subunit (NR2) (Ballif et al., 2003; Bock and Herz, 2003; Strasser et al., 2004; Chen et al., 2005; Mota et al., 2014). However, synaptic strength is determined by the ratio of NMDAR subunits (NR2A/NR2B) (Quinlan et al., 2004; Lebel et al., 2006; Brigman et al., 2008; Levenson et al., 2008). In this study, the level of NR2A and NR2B subunits and the estimated NR2A/NR2B ratio were higher in BME and 5-azaC groups compared to control. In addition, the co-immunoprecipitation studies further substantiate our hypothesis and showed enhanced interaction of NR2A subunit with SFK and PSD-95 in BME and 5-azaC groups compared to control group. This complex possibly enhances the Ca^2+^ influx through the release of glutamate, and then induces LTP and LTM (Chen et al., 2005; Qiu et al., 2006). Further, the activated NMDAR is in turn found to mediate events associated with methylation of BDNF and its subsequent regulation of transcripts and translation in the hippocampus during LTM. We found that level of unmethylated BDNF DNA was higher in BME and 5-azaC groups compared to control. Subsequently, the level of Bdnf (exon IV) mRNA and protein were found to be higher in BME and 5-azaC groups compared to control. There could be a possible correlation of unmethylated BDNF with its expression. However, we did not find a significant difference in the level of methylated form (Martinowich et al., 2003; Munoz et al., 2010). Interestingly, we observed a positive correlation between DI with unmethylated BDNF DNA in BME/5-azaC group. BME/5-azaC possibly regulate the exon specific expression by controlling methylation process (Sales et al., 2011), and the expression of BDNF in hippocampus enhances memory (Bekinschtein et al., 2007, 2008a,b; Lubin et al., 2008; Mizuno et al., 2012).

Taken together, we have shown the effect of BME in the regulation of DNA methylation using known *reelin-*dependent NMDAR–BDNF signaling. Our results show that the BME treatment dynamically controlled the methylation of *reelin* and subsequent alternative splicing of *ApoER 2*. Further, it might be implicated in the activation and interaction of NMDAR with synaptic protein (PSD-95) to induce BDNF. This mechanism might contribute to the modulation of synaptic plasticity and thus to enhance learning and memory.

## Author Contributions

Conceived and designed the experiments: KER. Performed the experiments: JP. Analyzed the data: KER and JP. Contributed reagents/materials/analysis tools: KER and HS. Wrote the paper: KER and JP.

## Conflict of Interest Statement

The authors declare that the research was conducted in the absence of any commercial or financial relationships that could be construed as a potential conflict of interest.

## Supplementary Material

**TABLE S1 | Time spent (s) exploring the objects during training by each experimental group (control; BME; 5-azaC) individuals.**
