# Supplementary material for: Possible Involvement of Standardized Bacopa monniera Extract (CDRI-08) in Epigenetic Regulation of reelin and Brain-Derived Neurotrophic Factor to Enhance Memory
Source: Front Pharmacol. 2016 Jun 27;7:166. doi: 10.3389/fphar.2016.00166 (PMC4921742; doi:10.3389/fphar.2016.00166)
Supplement: Supplementary file 1 [file Table_1.DOC]

|  |  | **Training I (Time spent (s))** | | | | **Training II (Time spent (s))** | | | |
| --- | --- | --- | --- | --- | --- | --- | --- | --- | --- |
| **Animal No.** | **GROUP NAME** | **Object 1** | **Object 2** | **Object 3** | **Object 4** | **Object 1** | **Object 2** | **Object 3** | **Object 4** |
| 1 | CON | 17 | 15 | 27 | 20 | 15 | 14 | 14 | 8 |
| 2 | CON | 26 | 16 | 11 | 19 | 23 | 9 | 3 | 27 |
| 3 | CON | 15 | 11 | 14 | 18 | 15 | 6 | 14 | 11 |
| 4 | CON | 15 | 18 | 15 | 23 | 12 | 6 | 9 | 14 |
| 5 | CON | 10 | 11 | 25 | 22 | 1 | 14 | 14 | 8 |
| 6 | CON | 4 | 0 | 9 | 10 | 6 | 4 | 13 | 7 |
| 7 | CON | 9 | 30 | 31 | 21 | 13 | 17 | 16 | 12 |
| 8 | CON | 9 | 10 | 20 | 8 | 13 | 37 | 15 | 21 |
| 9 | CON | 17 | 9 | 7 | 15 | 26 | 32 | 21 | 21 |
|  | Average | 13.56 | 13.33 | 17.67 | 17.33 | 13.78 | 15.44 | 13.22 | 14.33 |
|  | SD | 6.40 | 8.12 | 8.5 | 5.29 | 7.62 | 11.70 | 4.94 | 7.07 |
|  | SE | 2.13 | 2.70 | 2.83 | 1.76 | 2.54 | 3.90 | 1.64 | 2.35 |

|  |  | **Training I (Time spent (s))** | | | | **Training II (Time spent (s))** | | | |
| --- | --- | --- | --- | --- | --- | --- | --- | --- | --- |
| **Animal No.** | **GROUP NAME** | **Object 1** | **Object 2** | **Object 3** | **Object 4** | **Object 1** | **Object 2** | **Object 3** | **Object 4** |
| 1 | BME | 20 | 9 | 15 | 15 | 6 | 10 | 10 | 6 |
| 2 | BME | 8 | 19 | 17 | 18 | 13 | 7 | 10 | 22 |
| 3 | BME | 11 | 17 | 26 | 23 | 9 | 17 | 26 | 13 |
| 4 | BME | 19 | 33 | 35 | 11 | 7 | 35 | 10 | 17 |
| 5 | BME | 23 | 14 | 5 | 11 | 18 | 16 | 22 | 13 |
| 6 | BME | 21 | 3 | 15 | 16 | 7 | 15 | 17 | 24 |
| 7 | BME | 16 | 13 | 25 | 38 | 7 | 8 | 14 | 17 |
| 8 | BME | 17 | 7 | 12 | 6 | 26 | 11 | 21 | 20 |
| 9 | BME | 20 | 12 | 18 | 24 | 7 | 5 | 17 | 7 |
|  | Average | 17.22 | 14.11 | 18.67 | 18.00 | 11.11 | 13.78 | 16.33 | 15.44 |
|  | SD | 4.89 | 8.62 | 8.81 | 9.46 | 6.80 | 8.98 | 5.85 | 6.26 |
|  | SE | 1.63 | 2.87 | 2.93 | 3.15 | 2.26 | 2.99 | 1.95 | 2.08 |

|  |  | **Training I(Time spent (s))** | | | | **Training II(Time spent (s))** | | | |
| --- | --- | --- | --- | --- | --- | --- | --- | --- | --- |
| **Animal**  **No.** | **GROUP NAME** | **Object 1** | **Object 2** | **Object 3** | **Object 4** | **Object 1** | **Object 2** | **Object 3** | **Object 4** |
| 1 | 5-azaC | 17 | 20 | 41 | 43 | 16 | 6 | 19 | 14 |
| 2 | 5-azaC | 8 | 6 | 1 | 10 | 13 | 9 | 11 | 32 |
| 3 | 5-azaC | 10 | 6 | 24 | 29 | 5 | 10 | 12 | 34 |
| 4 | 5-azaC | 9 | 11 | 16 | 17 | 24 | 11 | 28 | 19 |
| 5 | 5-azaC | 1 | 9 | 3 | 7 | 4 | 6 | 8 | 0 |
| 6 | 5-azaC | 5 | 13 | 4 | 4 | 11 | 4 | 0 | 10 |
| 7 | 5-azaC | 0 | 31 | 0 | 0 | 12 | 2 | 0 | 10 |
| 8 | 5-azaC | 13 | 9 | 15 | 6 | 10 | 27 | 13 | 5 |
| 9 | 5-azaC | 16 | 6 | 11 | 14 | 6 | 8 | 4 | 6 |
|  | Average | 8.77 | 12.33 | 12.77 | 14.44 | 11.22 | 9.22 | 10.55 | 14.44 |
|  | SD | 6.03 | 8.30 | 13.30 | 13.68 | 6.22 | 7.25 | 9.05 | 11.83 |
|  | SE | 2.01 | 2.76 | 4.43 | 4.56 | 2.07 | 2.41 | 3.01 | 3.94 |
